# Prospects and challenges of recombinant spider venom enzymes: insights from *Loxosceles* and *Phoneutria* venom protease expressions

**DOI:** 10.3389/fbioe.2025.1668774

**Published:** 2025-09-19

**Authors:** Josephine Dresler, Ivonne Rodriguez, Anne Paas, Alfredo Cabrera-Orefice, Andreas Vilcinskas, Tim Lüddecke

**Affiliations:** ^1^ Fraunhofer Institute for Molecular Biology and Applied Ecology IME, Department for Bioresources, Giessen, Germany; ^2^ LOEWE Centre for Translational Biodiversity Genomics (LOEWE TBG), Frankfurt, Germany; ^3^ Institute of Biochemistry, Medical Faculty, Justus Liebig University, Giessen, Germany; ^4^ Institute for Insect Biotechnology, Justus Liebig University of Giessen, Giessen, Germany

**Keywords:** biotechnology, biodiscovery, enzyme technologies, metalloprotease, *Escherichia coli*, spider venomics, refolding, purification

## Abstract

Spiders use chemically complex venoms to overpower prey. Such venoms are primarily composed of neurotoxic disulfide-rich peptides, linear peptides, and enzymes. The latter have received little scientific attention thus far, and despite their great translational potential, functional data on spider venom enzymes remain scarce. Hence, a more comprehensive understanding is sought, not only to provide valuable insights into their biological functionality but also to facilitate the development of novel biotechnological applications. However, their chemical isolation is prevented by the minuscule venom yields available from most spiders. Recombinant expression emerged as a promising methodology to overcome these restrictions, but comparatively few efforts have been made to establish technologies for different enzyme families. In particular, few works have explored the pivotally important technical aspects of spider venom enzyme expression, including strain selection, culture conditions, and product purification. In this study, we explore these aspects using two spider venom enzymes as a case study, with particular emphasis on the purification and refolding of an astacin-like metalloprotease from *Loxosceles intermedia* venom. The enzymes were produced as fusion proteins using diverse *Escherichia coli* strains to identify the most effective production strains, including their optimal production conditions. Thioredoxin A, a 6x-His-Tag, and a cleavage site for activated factor X allowed efficient purification and subsequent removal of all fusion tags, and we report in detail the purification of the mass spectrometry-confirmed *L. intermedia* metalloprotease from inclusion bodies. This exploratory study outlines the technical details and potential pitfalls encountered during the development of this production process and provides an important baseline for future attempts to express spider venom enzymes.

## 1 Introduction

Spiders have evolved into highly effective predators that use potent venom to subjugate prey, primarily insects (Langenegger et al., 2019; Lüddecke et al., 2022). Their venom comprises up to 3,000 different biomolecules, the majority of which are disulfide-rich neurotoxic peptides, linear peptides, and proteins (Pineda et al., 2020). In particular, disulfide-rich venom peptides with an inhibitor cystine knot (ICK) motif have been the focus of various biomedical studies due to their interaction with the central nervous system (Vassilevski et al., 2009; Saez et al., 2010; Kuhn-Nentwig et al., 2011; Windley et al., 2012; Langenegger et al., 2019).

However, recent works suggested that high-molecular-weight components are also important elements of many spider venom systems (Kuhn-Nentwig et al., 2019; Zobel-Thropp et al., 2019; Lüddecke et al., 2020; Dresler et al., 2024a; 2024b). Among these, enzymes are of particular relevance because of their ability to catalyze numerous chemical reactions, but at the time of writing, little is known about their biological roles and functions (Dresler et al., 2024a; 2024b). Hence, their detailed characterization and functional interrogation will undoubtedly increase our understanding of the venom biology of spiders (Herzig, 2023). Moreover, the diversity of spider venom enzymes identified to date includes a large array of enzyme classes with proven bioeconomic potential (Dresler et al., 2024a; 2024b). Therefore, they offer novel translational avenues beyond biomedicine and agriculture. In particular, this comprises a wide range of hydrolases, which feature the most predominant group of spider venom enzymes (Dresler et al., 2024a; 2024b). For instance, chitinases degrade chitin and may be utilized for waste degradation in insect farming-based circular bioeconomy, while lipases hydrolyze various lipids and may be applied as detergents or in food and feed production. A large fraction of hydrolases consists of proteases from various families, including serine proteases and metalloproteases such as neprilysins and astacins. These proteolytic enzymes may be utilized for a multitude of applications, including food and feed production, protein modification, or as diagnostic tools. Nevertheless, although more comprehensive knowledge of spider venom enzymes is pivotal for a better understanding of spider venom biology and their bioeconomic exploitation, little research has been devoted to them, and despite few pioneering studies, virtually nothing is known about these compounds (Dresler et al., 2024a; 2024b). Closing this persistent gap in the scientific literature has recently been coined as a key question in arachnid toxinology (Herzig, 2023).

One of the major challenges when working with spider venoms is the procurement of venom compounds in adequate quantities for characterization (Sequeira et al., 2017). Although modern venomics technologies have facilitated the compositional analysis of venoms from even the smallest spiders, their chemical isolation remains hindered by the limited quantities available (von Reumont et al., 2014). An important strategy to overcome these limitations is the laboratory-scale production of selected biomolecules. In this framework, various synthetic biology approaches have been explored, and recombinant expression has emerged as the most effective approach for larger proteins such as spider venom enzymes (Stráner et al., 2016; Lüddecke et al., 2021; 2023; Rivera-de-Torre et al., 2022; Wu et al., 2022; Paas et al., 2024). The advent of recombinant protein production has led to the development of various prokaryotic and eukaryotic *in vivo* expression systems and even cell-free technologies. However, the prokaryotic expression system using *E. coli* remains the preferred host for recombinant expression as it is highly efficient, inexpensive, and robust (Francis and Page, 2010). The primary constraint on bacterial systems pertains to their inability to execute eukaryotic post-translational modifications (Francis and Page, 2010). However, spider venom enzymes, with very few exceptions, possess only disulfide bridges, the formation of which can be facilitated by various engineered *E. coli* strains (Francis and Page, 2010; Rivera-de-Torre et al., 2022). Additionally, these systems have demonstrated notable scalability to industrial levels, thus underscoring their relevance for large-scale applications (Francis and Page, 2010). Recombinant protein production has revolutionized toxinology as it has dramatically expanded the number of proteins that can be investigated (Francis and Page, 2010). However, compared with other venom components, only very few works to date have attempted to express recombinant spider venom enzymes, and these previous works have particularly focused on spider species and enzymes of clinical relevance. For instance, the recombinant production of venom hyaluronidases from brown spiders (genus *Loxosceles*) and a tarantula (*Brachypelma vagans*) has been explored (Clement et al., 2012; Ferrer et al., 2013; De-Bona et al., 2021), as has the production of phospholipase D from several species of brown spiders (Kalapothakis et al., 2002; Da Silveira et al., 2006; 2007a; Appel et al., 2008; Catalán et al., 2011; Lajoie et al., 2013; Magalhães et al., 2013). Furthermore, the recombinant production and characterization of astacin-like metalloproteases from *Loxosceles intermedia* has been reported (Da Silveira et al., 2007b; Morgon et al., 2016).

Although previous studies have focused on characterizing specific spider venom enzymes, the vast remaining diversity remains unexplored. This gap in knowledge is particularly concerning in light of the potential applications of spider venoms. A comprehensive understanding of the full spectrum of spider venom enzymes is essential for unveiling their biological relevance and harnessing their unique properties in translational applications. However, without a robust methodology for expression and screening, the efficacy of research efforts is impeded. Therefore, it is necessary to establish a reliable recombinant enzyme expression system that addresses the challenges inherent in spider venom enzyme production. The importance of technical details in the development of a production process cannot be underestimated, particularly as these enzymes are often produced in complex biological systems (i.e., venom glands) that facilitate precise modulation of multiple parameters, including precursor maturation and folding.

Each step in the recombinant production process, from gene cloning to purification, hence necessitates careful consideration to avoid pitfalls that could compromise the functionality and stability of the enzymes and impact downstream applications. Moreover, a comprehensive understanding of these technical nuances enables researchers to design more efficient screening systems, thereby accelerating the identification of promising enzyme candidates for further study. In this context, the present study uses a systematic approach to evaluate spider venom proteases (metalloprotease and serine protease), using various *E. coli* strains and expression conditions. By exploring multiple factors influencing the production success, this study aims to explore alternatives to facilitate the successful production and purification of expressed proteins. Hence, it aims to identify optimal settings for producing each of the selected enzymes as fusion proteins alongside lessons and pitfalls observed during their processing and purification. In particular, the challenges encountered during expression, including the formation of inclusion bodies and the need for effective refolding techniques, are meticulously outlined. By addressing these technical details, we aim to lay the groundwork for future innovations, paving the way for breakthroughs in both fundamental research and practical applications of spider venom enzymes.

## 2 Methods

### 2.1 Plasmid construction and production

**FIGURE 1:**
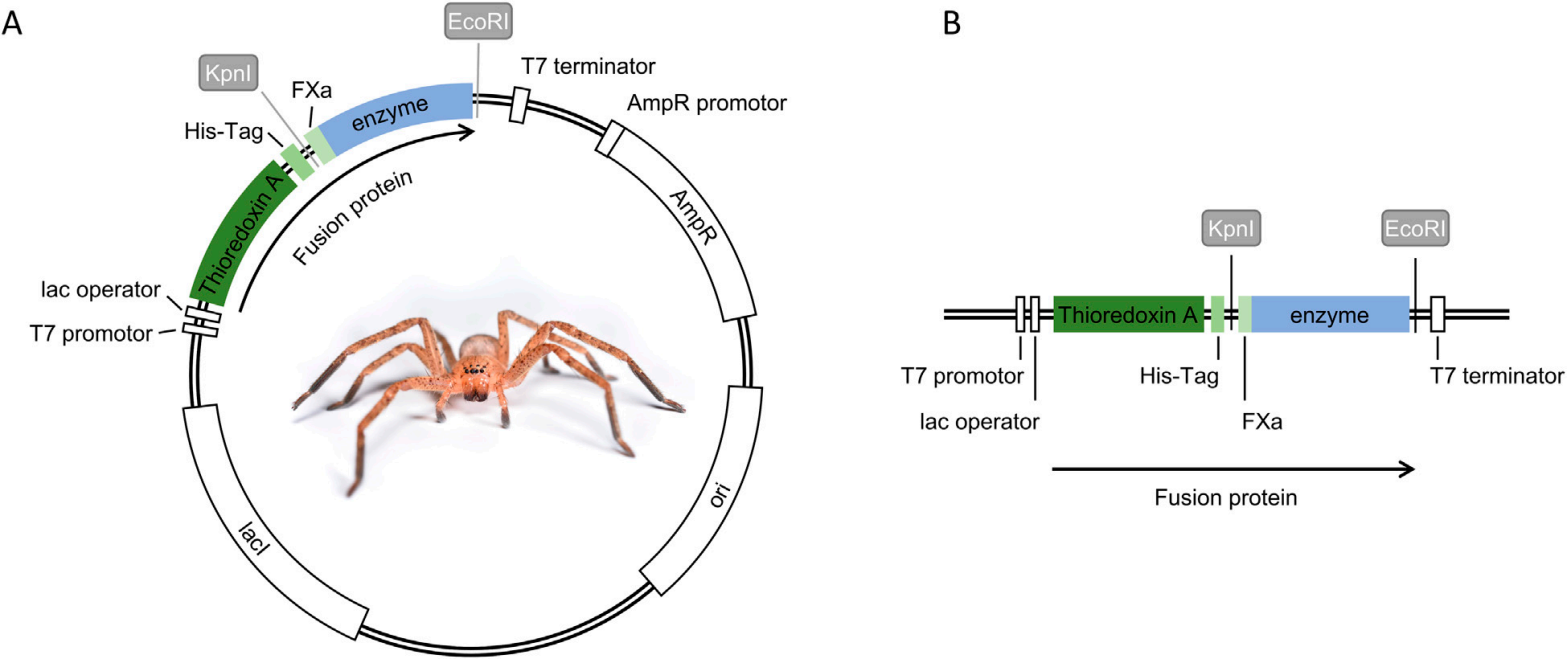
Expression plasmid construction for fusion protein production. **(A)** Expression plasmid map pET32a (+) including the fusion protein with all its features, **(B)** expression cassette of the fusion protein controlled by the T7 promoter, featuring thioredoxin A as the fusion partner, a 6x-His-Tag (His-Tag), Factor Xa cleavage site (FXa), and the selected spider venom enzyme (enzyme). Restriction enzyme sites (KpnI and EcoRI) were used to insert FXa and enzyme sequences.

Enzyme sequences were obtained by retrieving the corresponding domain sequence from UniProt [serine peptidase *Phoneutria nigriventer* P84033 (Phoni) and astacin-like metalloprotease *L. intermedia* C9D7R2 (Loxin)]. Translation and codon optimization for *E. coli* were carried out using EMBOSS Backtranseq by EMBL-EBI (Madeira et al., 2024). To enable subsequent Gibson cloning, 20 base pairs matching the vector sequence were added at the 3′ and 5′ ends during synthesis. Codon-optimized genes were synthesized using Thermo Fisher GeneArt Gene and Protein Synthesis Services (Germany), resulting in the delivery of Loxin as a gene fragment, whereas the gene fragment for Phoni was delivered within a plasmid. The *E. coli* expression vector pET-32a (+) (Novagen, Germany) and the plasmid containing the gene fragment of Phoni were prepared through digestion with EcoRI-HF and KpnI-HF restriction enzymes (New England Biolabs, Germany). The resulting opened vector and desired Phoni gene fragment were purified using the DNA recovery kit (Zymoclean Gel DNA Recovery Kit; Cat. No.: D4007, Zymo Research, Germany), according to the manufacturer’s instructions. For Loxin, the obtained enzyme sequence was transferred to the opened expression vector pET-32a (+) using the Gibson Assembly Cloning Kit (Cat. No. E5510S, New England Biolabs, Germany), following the manufacturer’s instructions. The gene fragment of Phoni was transferred to the open vector by ligation using a T4 DNA Ligase (Promega, M179A) in a vector: insert ratio of 1:3, according to the manufacturer’s instructions. The resulting plasmid (Figure 1) was used to transform chemically competent *E. coli* NEB® 5-alpha cells (Novagen, Germany) by mixing 2 µL plasmid with 25 µL competent *E. coli* NEB® 5-alpha cells. After a 15-min incubation on ice, a 45-s heat shock at 42 °C, followed by a 2-min incubation on ice, was carried out. A volume of 250 μL of the included SOC-medium was added to the sample and incubated for 1 h at 37 °C and 300 rpm agitation (Thermoblock, Eppendorf, Germany). Afterward, the cells were plated out on Luria-Bertani (LB) agar plates containing 100 μg/mL ampicillin (LB_amp_) and incubated overnight at 37 °C. One clone was used to inoculate 3 mL liquid LB_amp_ medium and incubated overnight at 37 °C and 180 rpm. The plasmid was purified using the NucleoSpin® Plasmid EasyPure Kit (Cat. No. 740.727.250, Macherey-Nagel GmbH & Co. KG, Germany), following the manufacturer’s instructions. DNA concentration and purity were determined photometrically with A_260_/_280_ using the BioTek® EON^TM^ Microplate Reader (BioTek Instruments, United States), and the plasmid sequence was confirmed using Oxford Nanopore sequencing (Eurofins Genomics, Germany).

### 2.2 Transformation of selected *E. coli* expression strains

Purified plasmids were transformed into six different chemically competent *E. coli* strains [Origami^TM^ 2, Origami^TM^ B, BL21 (DE3), Rosetta-gami^TM^ B, and Rosetta-Gami^TM^ 2 from Merck Millipore/Novagen (Germany) and SHuffle T7 Express lysY from New England Labs (Germany)]. For transformation, 10 ng of plasmid DNA was added to 100 µL chemically competent cells, and the procedure was carried out as described in Section 2.1. Afterward, cells were plated out on LB_amp_ agar plates and incubated overnight at 37 °C. One clone per successfully transformed strain was used to inoculate 3 mL of Terrific Broth (TB) medium with 100 μg/mL ampicillin (TB_amp_) and incubated at 37 °C and 180 rpm overnight. The culture was used to prepare cryo-conserved stocks by combining 500 µL of culture with 500 µL of 30% glycerol and stored at −80 °C.

### 2.3 Heterologous expression of the fusion proteins

Fusion protein production was examined by cultivating transformed *E. coli* strains using three distinct production temperatures. Fusion protein biosynthesis was confirmed using one-dimensional sodium dodecyl sulfate polyacrylamide gel electrophoresis (SDS-PAGE). This technique enabled the comparison of the expected protein bands with the present bands (Table 1). For this, overnight cultures of 3 mL TB_amp_ medium with 3 µL cryo stock were incubated at 37 °C and 180 rpm and used to inoculate 50 mL TB_amp_ medium in 250 mL baffled flasks. The cultures were incubated at 37 °C and 180 rpm until the bacterial growth reached a optical density measured at 600 nm (ΔOD_600_) of 0.8–1.0. Isopropyl-β-D-thiogalactopyranosid (IPTG) was added to a final concentration of 1 mM to induce the fusion protein biosynthesis under the control of a T7 promoter. After induction, cultures were incubated at three distinct temperatures (16 °C, 28 °C, or 37 °C). Cultures were incubated at 37 °C, 28 °C, and 16 °C were then incubated for 4 h, 16 h, and 24 h, respectively, and samples for SDS-PAGE were collected throughout the incubation period. For this, ΔOD_600_ was measured, and a cell volume corresponding to 500 μL cell culture/ΔOD_600_ per sample was collected. This standardized cell concentrations, enabling accurate estimation of protein yield without interference from variations in cell density. Cultivation medium was removed by centrifugation at 12,000 × g for 5 min, and the pellet was stored at −20 °C until analysis using SDS-PAGE.

**TABLE 1:** Target spider venom enzymes.

Enzyme family	Origin	Name	Fusion protein			Target enzyme			
			n AA	MW	pI	n AA	MW	pI	-S-S-
Metalloprotease	*Loxosceles intermedia*	Loxin	366	40.7	7.7	204	23.5	9.0	2
Serine protease	*Phoneutria nigriventer*	Phoni	407	44.0	6.6	245	26.8	8.1	3

Number of amino acids (n AA), molecular weight (MW) in kDa, and the isoelectric point (oI) of the fusion protein and the targeted enzyme, respectively. For the target enzymes, the number of disulfide bonds (-S-S-) is also included.

For the SDS-PAGE, the sampled pellets were resuspended in 25 µL ultra-pure water and 25 µL 2× Laemmli buffer with 6% β-mercaptoethanol. Afterward, samples were incubated at 95 °C for 5 min and centrifuged for 3 min at 12,000 × g. An aliquot of 3 μL of each sample, together with Precision Plus Protein™ Dual Xtra Prestained Protein Standards (Cat. No. 1610377, BioRad, Germany), was loaded onto a 4%–20% Criterion™ Tris-HCl Protein Gel (Cat. No. 3450034, BioRad, Germany), and gel electrophoresis was carried out with 150 V over 55 min. The gels were stained using Roti® Blue quick ready-to-use 1X solution (Carl Roth, Germany) for 2.5 h and destained with 10% ethanol to visualize protein bands on the gel.

### 2.4 Western blot

Expression samples were separated by reducing SDS-PAGE and transferred to a polyvinylidene difluoride (PVDF) membrane (Cat. No. 1704157, BioRad, Germany) using the Trans-Blot® Turbo™ Transfer System (BioRad, Germany). The membrane was blocked overnight at room temperature in 5% BSA solution, washed thrice with PBS +0.1% Tween 20, and incubated for 2 h at room temperature with Penta-His-HRP Conjugate (1:2000 dilution) and Precision Protein StrepTactin HRP (1:10000). Afterward, the membrane was washed thrice with PBS +0.1% Tween 20. Visualization was carried out using the Opti-4CN Substrate Kit (Cat. No. 1708235, BioRad, Germany).

### 2.5 Production and purification of Loxin fusion protein in *E. coli* BL21 (DE3)

As *E. coli* BL21 (DE3) was identified as the optimal production strain, fusion protein production was scaled up to 400 mL. For this, 3 mL of TB_amp_ medium was inoculated with 10 µL of cryo-conserved stock at 37 °C and 180 rpm. After 7 h of incubation, 100 µL of the culture was transferred into 50 mL of TB_amp_ medium in a 250-mL baffled flask and incubated overnight under the same conditions. To produce the fusion protein six times, 400 mL TB_amp_ medium in 1 L baffled flasks was inoculated with each 1 mL overnight culture. Bacterial growth was monitored using ΔOD_600_, and protein production was induced with 1 mM IPTG when cultures reached a ΔOD_600_ value of 0.8–1.0. After induction, the cultures were incubated at 28 °C for 5 h, and the cells were harvested by centrifugation for 15 min at 10,000 × g at 4 °C. The supernatant was discarded, and the cell pellet was stored at −20 °C until further processing. The cell pellet was resuspended with 100 mL lysis buffer (30 mM Tris and 100 mM NaCl; pH 7.5), and cell lysis was carried out using a Microfluidizer® (Microfluidics, United States) in two rounds. The lysate was centrifuged at 75,000 × g for 30 min at 10 °C; the resulting supernatant was discarded, and the pellet was stored at −20 °C. For solubilization, 10 g of wet pellet was suspended in 50 mL of solubilization buffer (8 M guanidinium chloride and 30 mM Tris; pH 7.5) and stirred overnight at room temperature. Afterward, the solution was centrifuged twice at 75,000 × g for 30 min at 10 °C. Due to the high viscosity, 9.5 mL of the solution was diluted with 240.5 mL solubilization buffer and applied to an immobilized metal affinity chromatography (IMAC) system (SE-04 FPLC) equipped with a 10 mL TALON® Superflow Resin Column (Cat. No. 635670, Clontech Laboratories, United States). The system was equilibrated with IMAC sample buffer (6 M urea, 30 mM Tris, and 100 mM NaCl; pH 7.5). After the sample application using 1 mL/min, the column was washed with IMAC sample buffer at a flow rate of 2 mL/min. Elution was performed at a flow rate of 1 mL/min using a stepwise gradient of 10%, 20%, 50%, and 100% IMAC elution buffer (6 M urea, 30 mM Tris, 100 mM NaCl, and 200 mM imidazole; pH 7.5) until baseline was reached. The process was monitored by UV absorbance at 280 nm, and fractions were collected for analysis by SDS-PAGE as described above.

### 2.6 Refolding of the recombinant Loxin fusion protein

Before refolding, the fusion protein (∼0.3 g/L) was supplemented with 1% β-mercaptoethanol and incubated at 37 °C for 20 min. Refolding was carried out by dialysis using a Roth dialysis tube with a 14 kDa cut off (Cat. No. 1784.1, Carl Roth). For this, several dialysis approaches were compared. Dialysis was carried out at 4 °C with continuous stirring using cold dialysis buffer at a ratio of 2 L buffer per 10 mL sample. After 18 h of incubation, the buffer was exchanged, and a second round of dialysis against fresh dialysis buffer was conducted for an additional 18 h. The solution was clarified by centrifugation (18,000 × g, 4 °C, 30 min). For the first dialysis approach, the same buffer was used for both rounds. For the second and third approaches, three rounds with different buffers were applied (Supplementary Table S1).

The first dialysis approach used Tris–NaCl buffer (20 mM Tris and 150 mM NaCl) at different pH levels (7.0, 7.5, 8.5, and 9.0) and tested initial fusion protein concentrations of 0.3 g/L, 0.2 g/L, and 0.1 g/L. Additionally, HEPES buffer (20 mM HEPES buffer, pH 6.8) and MES buffer (20 mM MES buffer, pH 6.5) were tested. The second dialysis approach used a Tris–NaCl buffer at pH 8.5, supplemented with 0.8 M L-arginine as the first buffer, 0.1 µM zinc sulfate as the second buffer, and no supplements as the third buffer. The third dialysis approach explored a Tris buffer (50 mM Tris, pH 8.5) following the same addition of L-arginine, zinc sulfate, or none as described in the second dialysis approach.

### 2.7 Surface adsorption of the recombinant Loxin fusion protein

The Loxin fusion protein showed surface adsorption after refolding, leading to a loss of protein. Therefore, two storage tubes and two detergents, commonly used for membrane proteins, were analyzed for their ability to prevent the attachment processes. For this, 20 µL aliquots of dialyzed Loxin fusion protein were transferred into either a low-binding tube (Sarstedt AG & Co. KG, Germany) or an HPLC glass vial (Fisher Scientific GmbH, Germany) and incubated. Additionally, Tween 20 or Triton X-100 was added directly to the dialysis tube to final concentrations of 0.8 mM or 0.5 mM, respectively. The mixtures were thoroughly mixed, and the solutions were transferred into low-binding tubes (Sarstedt AG & Co. KG, Germany). Samples were collected before transfer into the storage tube (S), immediately after transfer into the storage tube (t0), and after 20 h at 15 °C and 300 rpm (t1). Surface adsorption was explored by removing the solution and rinsing the tubes with 4 µL PBS buffer (PBS) and 8 µL Laemmli SDS buffer (SDS). Samples for SDS-PAGE analysis were prepared by combining 4 µL of the sample with 4 µL of 2× Laemmli and 6% β-mercaptoethanol. Samples obtained through the process of rinsing with Laemmli SDS buffer were collected without additional processing. Sample incubation and SDS-PAGE analysis were carried out as described before.

### 2.8 Cleavage of the recombinant Loxin fusion protein using activated factor X

To separate the fusion partners from the target enzyme Loxin, the fusion protein contains a cleavage site for activated factor X (FXa). Hence, the fusion protein was cleaved using FXa from bovine plasma (F9302, Sigma Aldrich, United States) in a 1:20 ratio (0.25 µg FXa +5 µg fusion protein) in FXa cleavage buffer (10 mM TRIS and 2 mM CaCl_2_; pH 8.0) at 15 °C and 300 rpm (Thermoblock, Eppendorf). To compare cleavage success, Loxin fusion protein with and without supplemented Tween 20 (0.8 mM) was incubated for 6 and 14 days, respectively. Evaluation of the cleavage was conducted using SDS-PAGE as described before.

### 2.9 Protease activity assay

Following the successful cleavage of the fusion protein, the activity of the target enzyme Loxin was analyzed as part of the development process. For this, refolded Loxin fusion protein from each of the three explored dialysis approaches (see Section 2.6/Section 3.5) was analyzed. In brief, refolded Loxin fusion protein supplemented with 0.8 mM Tween 20 was cleaved with 0.05 g FXa per g fusion protein for 48 h at 15 °C and 300 rpm. The protease activity was photometrically assessed using the non-specific Pierce Fluorescent Protease Assay Kit (Thermo Scientific, Germany), which utilizes FTC-casein as substrate. Loxin samples were analyzed without further processing, whereas 1 μg/mL trypsin and 4 μg/mL FXa (the corresponding concentration used for the digestion of the Loxin fusion protein) served as positive controls, and reaction buffer (25 mM Tris and 150 mM NaCl; pH 7.2) served as the negative control. After incubation for 1 h at room temperature, fluorescein excitation/emission at 485/538 nm with a gain of 50 was measured using the Synergy H4 Hybrid Microplate Reader (BioTek Instruments, United States).

### 2.10 Purification of Loxin

#### 2.10.1 Batch purification

After the successful cleavage of the fusion protein, further purification of the target enzyme Loxin was required. For this, fusion protein cleaved with FXa over 2 days at 15 °C and 300 rpm and supplemented with 0.8 mM Tween 20 was prepared and stored at −20 °C until usage. The purification efficiency of two chromatography methods was then evaluated.

Initially, a non-absorptive IMAC purification was conducted as a batch process. For this, TALON® Superflow Resin was utilized, which is stored in a 1:1 ethanol-to-resin ratio. The resin was prepared by resuspending it and washing 100 µL of resuspended material twice with 100 µL of ultra-pure water and once with IMAC buffer A (40 mM Tris and 400 mM NaCl, pH 8.0). Afterwards, the washed resin was resuspended in the corresponding buffer and centrifuged for 2 min at 12,000 × g, and the supernatant was discarded. The washed resin was then resuspended in 50 µL of IMAC buffer A, and 20 µL of prepared resin was used for the purification of 6 µg of target enzyme. For the purification of the target enzyme, raw FXa cleavage solution containing the cleavage products after FXa digestion was mixed with the resin. After a 2 min incubation and a 2-min centrifugation step, the supernatant was (flow-through) collected and stored for subsequent SDS-PAGE analysis. Subsequently, the resin was washed thrice with 20 µL IMAC buffer A (wash steps) and twice with 20 µL IMAC buffer B (elution steps, IMAC buffer A supplemented with 200 mM imidazole). In each step, the resin was resuspended in the relevant buffer, incubated for 2 min, centrifuged for 2 min, and the supernatant was stored for further SDS-PAGE analysis.

In the second examined chromatography process, cation exchange chromatography (CIEX) resin (BioPro IEX S75, YMC) was used at pH 8.0 and pH 6.0. Preparation was conducted as described above, using CIEX sample buffer (45.8 mM sodium acetate, pH 8.0/6.0) instead of IMAC buffer A and CIEX elution buffer (45.8 mM sodium acetate, pH 8.0/6.0 supplemented with NaCl) instead of IMAC buffer B. Purification was initiated by dilution of raw FXa cleavage solution containing 6 µg target enzyme in 1,000 µL CIEX sample buffer. This was subjected to incubation with 20 µL resuspended CIEX resin for 2 min. After centrifugation, the supernatant was discarded, and the resin was washed twice with 20 µL CIEX sample buffer. Elution was conducted in a stepwise manner by applying 20 µL of CIEX elution buffer containing 0.1 M, 0.2 M, 0.5 M, and finally 1 M NaCl. In each step, the resin was resuspended in the buffer, incubated, and centrifuged, and the supernatant was stored. The samples were analyzed using SDS-PAGE as described previously.

#### 2.10.2 Fast protein liquid chromatography

An NGC™ Chromatography System (Bio-Rad, Germany) equipped with a HiScreen Capto S column (Cytiva, United States) was used for CIEX, applying a linear elution gradient. For this, 1.5 mg Loxin fusion protein was refolded using the third dialysis approach and cleaved by incubation with FXa and Tween 20 for more than 48 h at 15 °C. To remove precipitates formed during cleavage, the solution was centrifuged at 10,000 × g for 30 min at 4 °C. The resulting sample was diluted 1:20 with CIEX sample buffer (45.8 mM sodium acetate, pH 6.0) and applied at a flow rate of 0.8 mL/min to the CIEX column. After the sample application, the column was washed with five column volumes of CIEX sample buffer, followed by a linear elution gradient (0%–100%) over 40 column volumes using CIEX elution buffer (45.8 mM sodium acetate and 1 M NaCl, pH 6.0) at a flow rate of 1 mL/min. The process was monitored by UV absorbance at 220 nm and 280 nm, and 1 mL fractions were collected for analysis by SDS-PAGE as described above.

### 2.11 Mass spectrometry

In-gel trypsin digestion followed by tandem mass spectrometry analysis was carried out for protein confirmation. For this, an SDS-PAGE was performed, and gel spots containing Loxin were cut out and fully destained through several washing steps using 50% methanol and 50 mM ammonium bicarbonate (ABC). To reduce and alkylate cysteine residues, the gel pieces were incubated in 10 mM TCEP, 40 mM chloroacetamide, and 50 mM ABC for 45 min at 500 rpm in the absence of light. After a shrinking step for 15 min in 50% methanol and 50 mM ABC, the gel pieces were air-dried under a laminar flow hood for 30 min. The protein was digested using 100 ng of sequencing-grade modified trypsin (Promega) dissolved in 70 µL of 50 mM ABC for 12 h at 37 °C, and the resulting peptides were collected in a clean PCR plate. To increase recovery, the gel pieces were incubated with 50% methanol and 1.5% formic acid (FA), and the remaining peptides were centrifuged and combined with the first flow through. The peptides were dried in a Concentrator Plus (Eppendorf, Germany) device for 3 h at 45 °C (V-AQ Mode) and finally resolubilized in 2% acetonitrile (ACN) and 0.1% FA.

Nano liquid chromatography and mass spectrometry (MS) were carried out using a Thermo Fisher Orbitrap Eclipse equipped with an ultrahigh-performance liquid chromatography unit Dionex UltiMate 3000 (Thermo Fisher, Germany), a Nanospray Flex Ion Source (Thermo Fisher, Germany), a C18 precolumn (Thermo Scientific, Germany), and a 1.9-µm C18 column (Aeros 25 cm × 150 µm ID, Protum*Link*, Germany). MS was carried out using a linear gradient from 2% ACN and 0.1% FA to 35% ACN and 0.1% FA for 40 min, followed by a gradient from 35% to 95% ACN in 3 min. The analytical column was washed with 90% ACN and 0.1% FA for 5 min and then equilibrated with 2% ACN and 0.1% FA for at least 5 min. The flow rates were set as 500 nL/min (first 45 min) and 800 nL/min (last 15 min). MS data were recorded through data-dependent acquisition. The full MS scan range was 375–1500 m/z with a resolution of 75,000, an automatic gain control (AGC) value of 3E6, and a maximal ion injection duration of 148 ms. MS1 data were acquired in profile mode. The 20 most abundant precursors (charge 2–7) were selected for MS/MS and detected using the ion trap (Rapid), with an isolation window of 1.6 m/z, an automatic gain control value set to 25,000, and a maximal ion injection duration of 35 ms.

FragPipe v23.0 (Kong et al., 2017; Yu et al., 2021) was used to perform the proteome search. N-terminal acetylation (+42.01) and oxidation of methionine (+15.99) were selected as variable modifications, and carbamidomethylation (+57.02) on cysteines was selected as a fixed modification. The list of common contaminants was supplemented manually with the sequence of the protein of interest used to identify peptides and proteins with a false discovery rate (FDR) less than 1%. The workflow “LFQ-MBR” was selected for the search, with all settings kept at default.

## 3 Results and discussion

### 3.1 Plasmid production

The expression in *E. coli* is influenced by multiple parameters that can be adapted when optimizing an expression protocol. These factors include the selection of the expression vector, the expression cell line, and the process conditions, among others.

In this study, we selected the *E. coli* expression vector pET-32a (+) that utilizes the T7 lacO promoter system and contains an N-terminal 6x-His-Tag, thioredoxin A, and a cleavage site for FXa (Figure 1). The T7 promoter grants control over the expression of the fusion protein, while 6x-His-Tags are commonly used to facilitate purification via affinity chromatography. Furthermore, thioredoxin A helps increase the solubility of expressed proteins, and the FXa cleavage site allows the proteolytic removal of all fusion partners, yielding the target enzyme without leaving non-native residues at the expressed protein of interest (Francis and Page, 2010). The domain sequence for the selected spider venom enzymes was cloned into the multiple cloning sites of the pET-32a(+) vector by Gibson cloning and ligation. Sufficient plasmid amounts were achieved through transformation of chemically competent *E. coli* NEB® 5-alpha cells. This approach was successful for all expression plasmids, as evidenced by plasmid production and subsequent confirmation through Oxford Nanopore sequencing (Supplementary Figures S1, S2).

### 3.2 Transformation of expression strains

To ascertain the most efficacious expression system, six *E. coli* strains were transformed with the generated expression plasmids. As outlined in Table 2, the transformation was successful for all *E. coli* strains with Loxin. In contrast, only Ori 2 and ST7 were transformed using the Phoni plasmid. Following cultivation on agar plates, a single clone from each construct was used to inoculate liquid medium. This resulted in the exclusion of RG B for Loxin due to the absence of observable growth in this environment.

**TABLE 2:** Transformation success of different *E. coli* expression strains. Overview of the transformation of the different *E. coli* strains with the expression plasmids for Loxin and Phoni and their subsequent growth on agar and in liquid LB containing 100 μg/mL of ampicillin.

*E. coli* strain	Loxin		Phoni	
	Plate	Liquid	Plate	Liquid
Ori 2	✔	✔	✔	✔
Ori B	✔	✔	✗	✗
BL21	✔	✔	✔	✔
RG 2	✔	✔	✗	✗
RG B	✔	✗	✗	✗
ST7	✔	✔	✔	✔

The symbol (✗) indicates a failed transformation or growth, whereas (✔) indicates a successful transformation and growth. *E. coli* expression strains: OrigamiTM 2 (Ori 2), OrigamiTM B (Ori B), BL21 (DE3) (BL21), Rosetta-gami^TM^ 2 (RG 2), Rosetta-gami^TM^ B (RG B), and SHuffle T7 Express lysY (ST7).

### 3.3 Fusion protein expression across hosts and temperatures

After successful transformation and cultivation in liquid media, the production of the fusion proteins was carried out at three different expression temperatures to identify the optimal production conditions. In particular, we compared fusion protein expression between 16 °C, 28 °C, and 37 °C. For this, each expression strain was cultivated at 37 °C until an ΔOD_600_ of 0.8–1.0, then induced with IPTG, and transferred to the corresponding expression temperature, and the presence of fusion proteins was validated through SDS-PAGE and Western blot analysis. Table 3 outlines the success of fusion protein expression at the tested temperatures across available expression strains for both constructs. Loxin fusion protein was successfully produced at all temperatures using all *E. coli* strains except Ori 2. Although RG 2 could be cultivated for the purpose of cryo-conservation stocks, its growth was severely reduced, which impeded induction. In contrast to the high transformation efficiency of Loxin, only Ori 2, BL21, and ST7 were successfully transformed using the Phoni plasmid, and protein production was detected in BL21 and ST7 at all temperatures.

**TABLE 3:** Fusion protein biosynthesis using diverse *E. coli* expression strains and conditions. Overview of fusion protein expression in 50 mL cultures, analyzed by SDS-PAGE using different *E. coli* strains as hosts.

*E. coli* strain	Loxin			Phoni		
	16 °C	28 °C	37 °C	16 °C	28 °C	37 °C
Ori 2	✗	✗	✗	✗	✗	✗
Ori B	✔	✔	✔			
BL21	✔	✔	✔	✔	✔	✔
RG 2	✔	✔	✔			
RG B						
ST7	✔	✔	✔	✔	✔	✔

The sign (✔) indicates the successful expression of the desired fusion protein, while (✗) indicates that no protein expression could be detected. Empty fields indicate that the experiment could not be conducted as no successful cloning of the plasmid in the strain was achieved or induction was impeded due to severely reduced cell growth.

### 3.4 Production and purification of the Loxin fusion protein

Based on the previous results concerning strain selection and suitable expression temperatures, we opted to henceforth focus on exploring avenues of production for Loxin as this protein appeared more promising in the experiments conducted to date. Therefore, following the successful production of the Loxin fusion protein, we proceeded with the development of subsequent purification steps.

**FIGURE 2:**
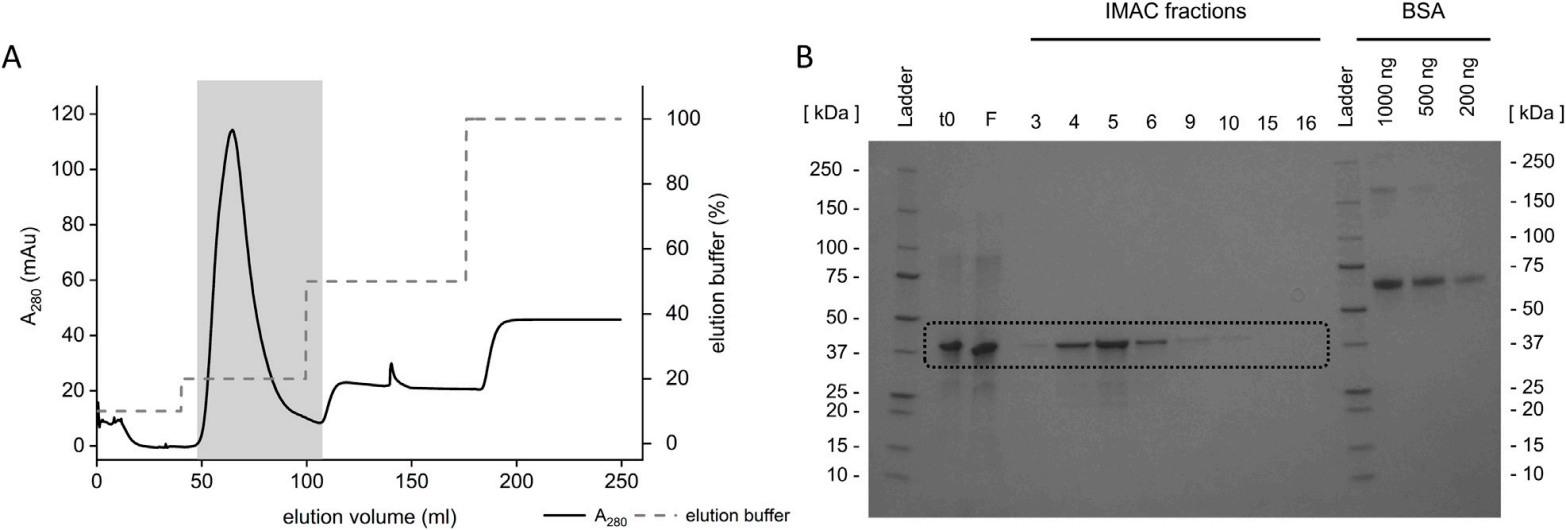
Affinity purification of the Loxin fusion protein. **(A)** Loxin fusion protein (∼41 kDa, B: black box) was produced in *E. coli* BL21 and purified using IMAC under denaturing conditions. The gray dashed line indicates the applied gradient of IMAC elution buffer. **(B)** SDS-PAGE displaying the Loxin fusion protein (t0), the IMAC flow-through (F), collected elution fractions, and a BSA protein reference. Fractions 4–8 from 48 to 110 mL (A: gray box) contained fusion protein.

SDS-PAGE analysis revealed that the fusion protein was produced as inclusion bodies under all tested *E. coli* strains and conditions (Supplementary Figure S3). Consequently, the optimal production conditions for Loxin were determined as expression at 28 °C over a duration of 5–6 h using BL21 due to the highest fusion protein yield (Table 3, Supplementary Figure S4). After cell lysis, the fusion protein was dissolved and purified using IMAC under denaturing conditions (Figure 2). The flow-through and collected IMAC fractions were analyzed by SDS-PAGE, which revealed the presence of the Loxin fusion protein in the flow-through and the first elution peak. Subsequent analysis indicated that the capacity of the IMAC resin had been exceeded, resulting in the presence of the fusion protein in the flow-through. The elution fractions 4–8 contained the Loxin fusion protein in an estimated concentration of approximately 0.3 g/L, resulting in a total yield of approximately 18.5 mg. As the obtained fusion protein required refolding, a range of dialysis approaches was subsequently explored.

### 3.5 Refolding

Refolding of the Loxin fusion protein was necessary because it was expressed as inclusion bodies; however, a portfolio of available refolding approaches for spider venom enzymes is virtually unavailable. Accordingly, we compared three different dialysis approaches to identify the most suitable protocol for refolding the Loxin fusion protein.

**FIGURE 3:**
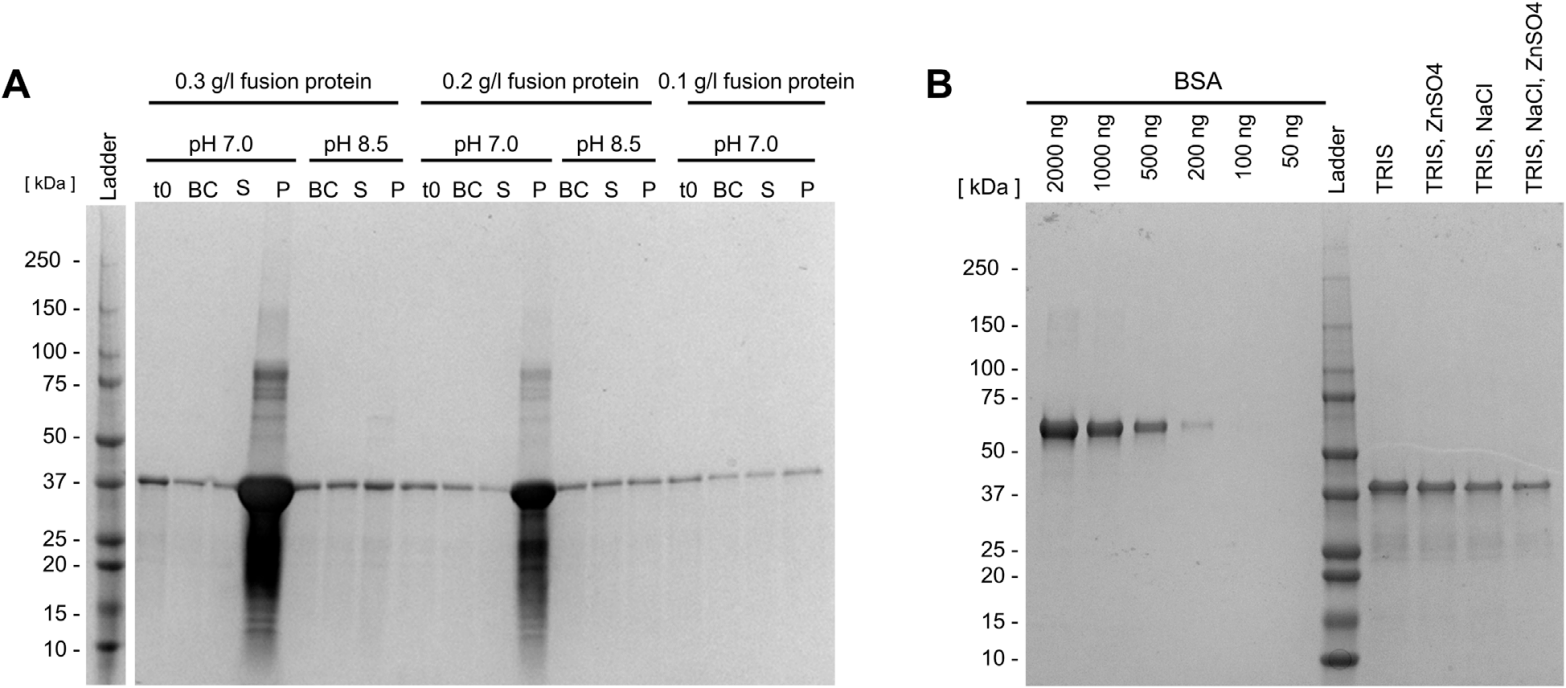
Refolding of the Loxin fusion protein (∼41 kDa) under different dialysis conditions. **(A)** The first dialysis approach (TRIS and NaCl) at pH 7.0 and 8.5 using different fusion protein concentrations led to the substantial precipitation of the fusion protein at pH 7.0.; S, supernatant (contains fusion protein); P, precipitated protein; t0, before refolding; BC, before centrifugation. **(B)** Second (Tris–NaCl buffers) and third (Tris buffers) dialysis approaches. SDS-PAGE analysis of the refolded fusion protein after dialysis using the second (ZnSO_4_) and third buffers (no supplement) including a BSA protein reference for concentration estimation. The initial protein concentration was 0.3 g/L, and the final protein concentration was ∼0.2 g/L (Tris and Tris–NaCl).

First, considering its theoretical isoelectric point (pI) of 7.7 and the pI of the natural enzyme (9.0), a Tris–NaCl dialysis buffer at different pH levels (7.5–9.0) and an initial protein concentration of 0.1 g/L were used. This revealed high precipitation at pH 7.5, as indicated by the absence of soluble fusion protein (Supplementary Figure S5A), while less precipitation was observed at pH 9.0 (Supplementary Figure S5B). The utilization of HEPES dialysis buffer and MES dialysis buffer at pH levels 6.8 and 6.5, respectively, also resulted in elevated precipitations, leading to no soluble fusion protein (Supplementary Figure S5C). Accordingly, both buffers were excluded from subsequent trials. Consequently, Tris–NaCl dialysis buffer at pH 7.0 or 8.5, with inital protein concentrations ranging from 0.1 g/L to 0.3 g/L revealed similar results (Figure 3A). The highest solubilized protein concentration was achieved using an initial concentration of 0.3 g/L fusion protein at pH 8.5.

To minimize precipitation, two additional approaches were explored. First, dialysis was carried out using a buffer containing L-arginine, which was subsequently exchanged for a second buffer containing zinc sulfate, followed by a buffer without supplements at pH 8.5. These approaches yielded no visible precipitation after the first dialysis step and only minuscule precipitation after the second and third steps. Figure 3B illustrates refolded Loxin fusion protein after the second and third dialysis steps, revealing increased refolding yields of estimated ∼0.2 g/L protein.

**FIGURE 4:**
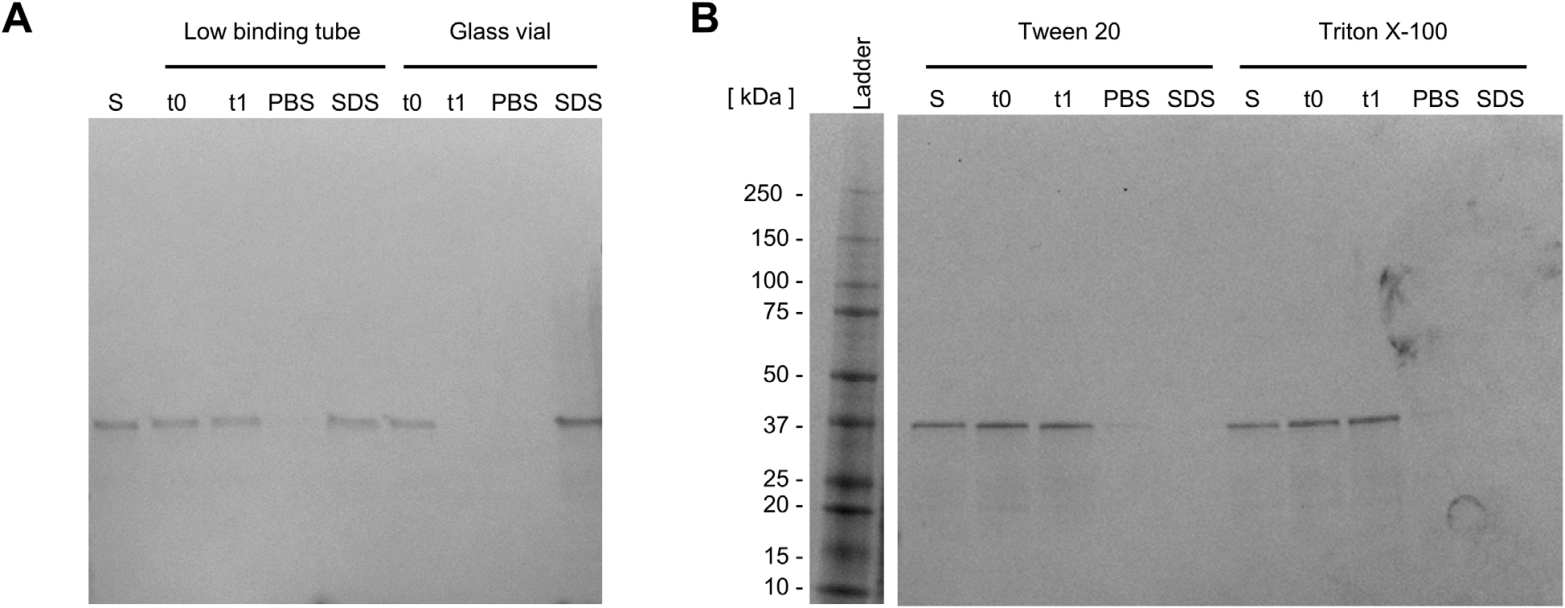
Preventing surface adsorption of the Loxin fusion protein. SDS-PAGE analysis of Loxin fusion protein, **(A)** in two storage tubes, **(B)** supplemented with 0.8 mM Tween 20 or 0.5 mM Triton X-100 to prevent surface attachment. Dialyzed fusion protein with/without the addition of detergents before transfer into tube (S), immediately after transfer (t0), and after 20 h of incubation (t1) at 15 °C and 300 rpm. After incubation, the solution was removed, and the tube was rinsed with PBS buffer (PBS) and Laemmli SDS buffer (SDS) to detect remaining fusion protein.

Following refolding, surface adsorption of the Loxin fusion protein was detected. This was evidenced by protein loss during storage, which may be attributed to the adsorption of the protein on the surface of the storage tube. To confirm this hypothesis, the storage tube was rinsed using PBS and Laemmli SDS buffer, and the samples were analyzed using SDS-PAGE. By rinsing with PBS, remaining protein traces were removed from the tube, while the SDS in Laemmli SDS buffer causes proteins to detach from surfaces by denaturation (Winogradoff et al., 2020). As protein bands could be observed after rinsing the tube with Laemmli SDS buffer, surface adsorption was verified. Hence, low-binding tubes and glass vials were evaluated for their potential to prevent protein adsorption (Figure 4A). The utilization of low-binding tubes revealed protein bands after rinsing with Laemmli SDS buffer (Figure 4A, lane SDS), while no soluble fusion protein could be detected in the glass vial (Figure 4A, lane t1 and SDS). Consequently, two detergents typically applied for membrane proteins—0.8 mM Tween 20 and 0.5 mM Triton X-100—were employed, and the surface attachment was examined. As illustrated in Figure 4B, the two detergents result in the formation of the solubilized fusion protein. This is evidenced by the absence of a protein band following the rinsing of the low-binding tube with PBS and Laemmli SDS buffer (Figure 4B, lanes PBS and SDS). As Tween 20 is known to increase enzyme stability and activity, it was selected for further experiments (Duskey et al., 2021).

### 3.6 FXa cleavage

**FIGURE 5:**
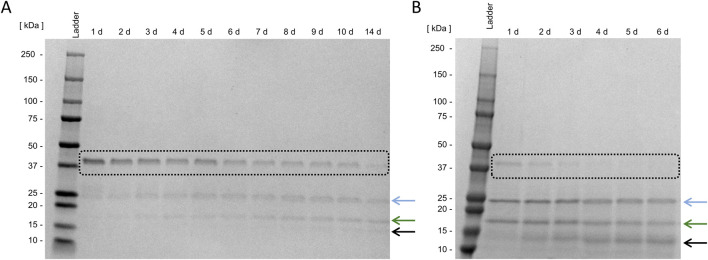
Fusion protein cleavage using activated Factor X. Digestion of 5 ng Loxin fusion protein (black box) using 0.25 ng activated factor X (FXa) without **(A)** and with 0.8 mM Tween 20 **(B)** at 15 °C. The blue arrow indicates the target enzyme Loxin, the green arrow indicates the fusion partner thioredoxin A, and the black arrow indicates an unknown digestion byproduct.

To separate the fusion partner thioredoxin A, including His-Tag and the FXa cleavage site, from the target enzyme Loxin, digestion with FXa is necessary. Figure 5 displays the cleavage of the Loxin fusion protein with and without Tween 20. The comparison reveals complete cleavage after 4 days of incubation with supplemented Tween 20, while in the absence of Tween 20, even after 14 days, no complete digestion was observed. Furthermore, SDS-PAGE analysis showed an additional protein band at ∼15 kDa, which is an unknown digestion byproduct possibly containing cleaved thioredoxin or Loxin (black arrow, Figure 5). This highlights the importance of preventing protein adsorption to surfaces as it appears to reduce the cleavage efficiency of FXa. Prior to the purification, the protease activity was photometrically assessed using the digestion products from each of the different dialysis approaches. In all cases, we detected activity ranging from 64% to 92% of the Loxin digestive approaches compared to the 1 μg/mL trypsin control, whereas the FXa control revealed 20% activity (Supplementary Figure S6). Thus, we concluded an activity of the Loxin enzyme.

### 3.7 Loxin purification

The purification of the target enzyme Loxin was investigated by comparing two distinct chromatography approaches to establish the most effective protocol for future studies.

**FIGURE 6:**
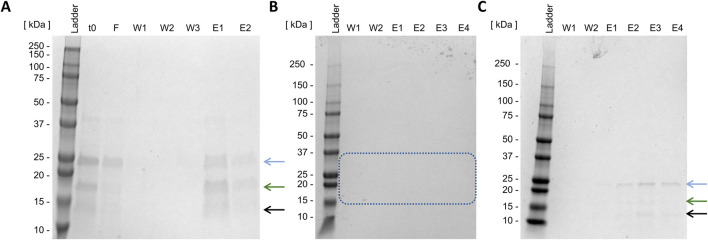
Batch Loxin purification using IMAC **(A)** and cation exchange chromatography **(B,C)**. t0, raw FXa cleavage solution; F, flow-through; W, wash steps; E, elution steps; **(A)** imidazol elution, **(B)** CIEX pH 8.0, stepwise elution with NaCl, the blue box indicates the absence of expected protein sizes; **(C)** CIEX pH 6.0, stepwise elution with NaCl. The blue arrow indicates the target enzyme Loxin (∼24 kDa), the green arrow indicates the fusion partner thioredoxin A with His-Tag and FXa cleavage site (∼17 kDa), and the black arrow indicates an unknown digestion byproduct (∼15 kDa).

Initially, non-absorptive IMAC was conducted. As exclusively the fusion partner contains a His-Tag, the target enzyme should elute with the flow-through (lane F, Figure 6A). As demonstrated in Figure 6, the flow-through contains Loxin (blue arrow). However, subsequent SDS-PAGE analysis of elution fractions also reveals protein bands corresponding to thioredoxin A (green arrow) and an unknown digestion byproduct (black arrow). Following three washing steps (lanes W1–W3, Figure 6A), elution with imidazole further revealed that the target enzyme had adsorbed to the resin (lanes E1 and E2, Figure 6A).

**FIGURE 7:**
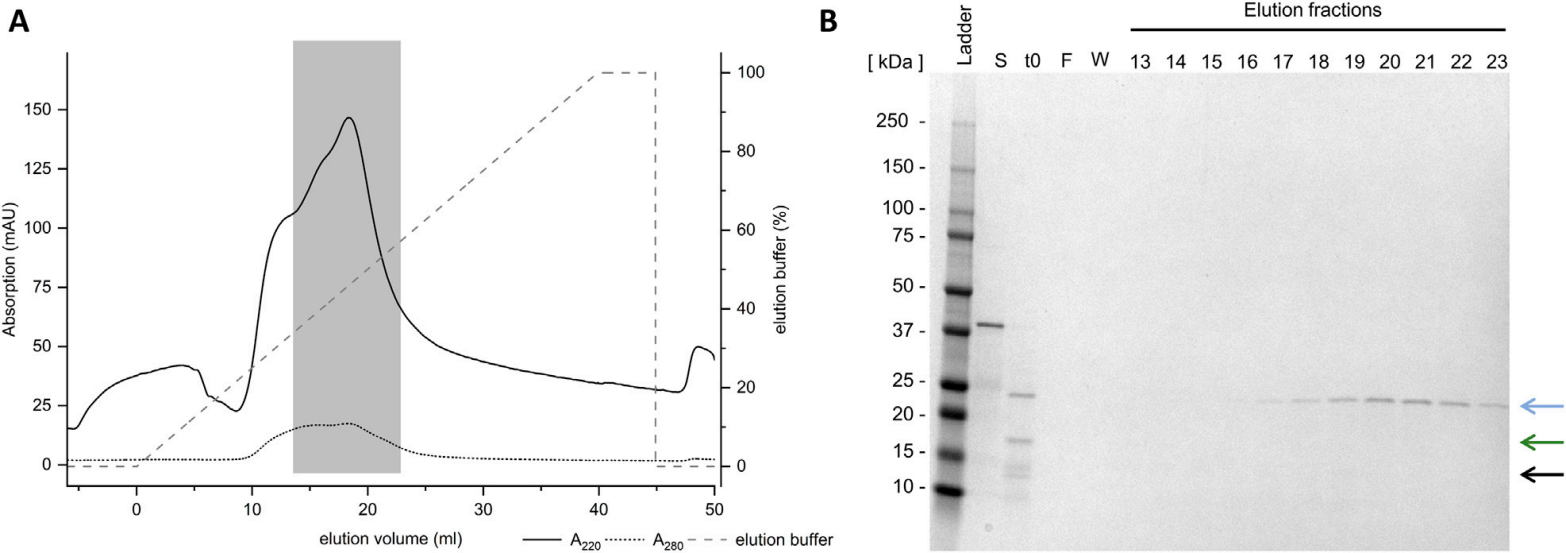
Purification of Loxin using cation exchange chromatography. **(A)** Loxin (∼25 kDa, blue arrow) was purified using CIEX at pH 6.0. The gray dashed line indicates the applied gradient of CIEX elution buffer. The gray box indicates the Loxin protein. **(B)** SDS-PAGE displays collected elution fractions, indicating the presence of Loxin in fractions 16–25, corresponding to elution volumes of 13.5 mL–22.8 mL. The blue arrow indicates the target enzyme Loxin (∼24 kDa), the green arrow indicates the fusion partner thioredoxin A with His-Tag and FXa cleavage site (∼17 kDa), and the black arrow indicates an unknown digestion byproduct (∼15 kDa).

As non-absorptive IMAC proved unsuccessful, CIEX was examined next. It is anticipated that this method will result in the charge-mediated binding of Loxin to the resin at different pH levels. CIEX was conducted at pH 8.0 and 6.0, which should result in a positively charged Loxin due to its pI of 9.0 while causing anionic charges on thioredoxin A (pI 4.7) and fusion protein (pI 7.7). The application of CIEX at pH 8.0 proved to be unsuccessful as no protein could be detected through SDS-PAGE analysis (Figure 6B, blue box). Conversely, the adjustment to pH 6.0 resulted in the elution of Loxin and an unknown digestion byproduct (Figure 6C). Consequently, another CIEX was conducted using a fast protein liquid chromatography system equipped with a HiScreen Capto S column at pH 6.0 and a linear elution gradient (Figure 7A). The analysis of CIEX fractions by SDS-PAGE (Figure 7B) demonstrated an elution of Loxin between 35% (=350 mM NaCl) and 60% (=600 mM NaCl) elution buffer. A total of 1.5 mg of fusion protein was prepared for this CIEX, of which approximately 0.3–0.5 mg of pure Loxin was recovered. This result is consistent with our calculations, which predict that 1.5 mg of fusion protein should yield approximately 0.9 mg of Loxin, of which a portion was observed to precipitate during the FXa cleavage. Finally, we used mass spectrometry to verify the identity of the purified protein. The identity of Loxin was confirmed by MS based in a bottom-up proteomics experiment, achieving 76% sequence coverage (Supplementary File S1).

### 3.8 Novel lessons on the expression of spider venom proteases

Spider venoms constitute a vast and largely untapped resource of novel enzymes with the potential to address fundamental biological questions and provide value for various biotechnological purposes. Enzymes already find application in pharmaceutical, analytical, and nutrition industries. Due to their high selectivity, low by-product formation, and eco-friendly characteristics, their utilization has rapidly increased (Singh et al., 2016; Industrial Enzymes Market, 2025). Venom enzymes, from spiders and other organisms, are active under challenging environmental conditions and often catalyze specific reactions. They, therefore, provide desired properties for industrial development (Lorenz and Eck, 2005; Industrial Enzymes Market, 2025). However, the diversity and biological significance of spider venom enzymes have only recently been revealed. Consequently, their characterization, which features a baseline to understand their biological significance and explore their translational value, remains pending.

Due to limited venom yields, recombinant expression emerged as the primary means to access these enzymes at the laboratory scale. However, compared to other venom components, the expression of spider venom enzymes remains largely underexplored. Particularly for unstudied biomolecules, the exploration of multiple factors needs to be considered during the development process toward a methodology for expression and screening. The features of the expression vector, such as the origin of replication and promoter, and protein properties, including the size and complexity, directly influence the transcription and translation of the target protein. Moreover, the choice of fusion partners has been demonstrated to have major effects on expression success and protein solubility but will, in turn, also modify the characteristics of the expressed protein (Klint et al., 2013; Nozach et al., 2013; Turchetto et al., 2017). Hence, in this study, we focused on the exploration and optimization of a recombinant expression workflow for spider venom enzymes using bacterial systems. In our study, the expression of an astacin-like metalloprotease from *L. intermedia* venom and a serine peptidase from *P. nigriventer* venom were explored as a case study. The major goal of this study was to evaluate various technical aspects of spider venom enzyme expression prior to functional characterization, which is an important but often neglected facet of venom biotechnology. As it stands, our data may be valuable to colleagues interested in characterizing spider venom enzymes and will contribute to a more efficient research environment. By investigating factors influencing the expression success, such as strain selection and expression conditions, this study may provide valuable alternatives to facilitate production, particularly when no target protein expression is initially observed. In particular, during our work, we encountered several pitfalls and issues on various process steps, which are meticulously documented and outlined hereafter.

#### 3.8.1 Suitability of the pET32a(+) vector

The first area of protein expression involves creating suitable vectors, designing suitable insert constructs, and transforming expression hosts.

To explore this, the codon-optimized domain sequences of our target enzymes were subcloned into the multiple cloning sites of the vector pET32a(+). This subcloning process revealed to be successful as all sequences were found correct after nanopore sequencing of the entire plasmid. The insert was strategically designed to create fusion proteins to ensure the inactive production of the enzyme, following biosecurity regulations in many countries. At the same time, the creation of fusion proteins allows the specific properties of the chosen fusion partner to be exploited to aid production. In our case, we designed fusion proteins, resulting in the spider venom enzyme merged with a 6x-His-Tag carrying thioredoxin A and an FXa cleavage site (Figure 1). The rationale behind this design was to leverage the chaperone-like capacities of thioredoxin A to aid in folding while facilitating purification following FXa cleavage without introducing unnatural residues (Francis and Page, 2010; Rivera-de-Torre et al., 2022). Although a variety of plasmids and expression strategies are available for spider venom components, our assessment showed that the commonly utilized pET32a(+) is effective for expressing spider venom enzymes by *E. coli*. This is exemplified by the high diversity of strains that were successfully transformed and verified to express both proteins. Overall, almost all strains were successfully transformed with Loxin, and half were transformed with Phoni (Table 1), and often, we recovered expressed fusion protein (Table 2). Therefore, it appears that the simple, commercially available pET32a(+) vector is a good starting point for spider venom enzyme studies. So far, previous works on the expression of spider venom components relied mostly on the pET-14b or pET-SUMO vectors. These vectors allow the addition of a 6x-His-Tag, including a thrombin recognition site or small ubiquitin-related modifier (SUMO), respectively (Ferrer et al., 2013; Morgon et al., 2016). Moreover, the utilization of self-cleaving elastin-like protein (ELP) tags could emerge as a promising approach. The ELP tag has been demonstrated to facilitate ELP-mediated precipitation and self-cleavage, thus allowing efficient purification of the target protein (Müller et al., 2015; Gao et al., 2018). With this, we herein add another potential vector to the available toolkit of spider venom biotechnology.

#### 3.8.2 Understanding the importance of diversity when probing expression systems and conditions

Once a suitable vector with the designed insert is created, the next step is the determination of the most promising expression system and the optimal expression parameters, especially focusing on the temperature. Although a broad range of potential expression systems is commercially available, it is not possible to choose the best system *a priori*, and multiple conditions should be evaluated. For instance, previously, spider venom enzymes (hyaluronidases, phospholipase D, and metalloprotease astacin) have been expressed using bacterial expression systems or a baculovirus–insect cell expression system (Da Silveira et al., 2007b; Clement et al., 2012; Ferrer et al., 2013; Lajoie et al., 2013).

Here, six *E. coli* strains were compared: *E. coli* BL21 (DE3) and its derivatives are most frequently used for routine protein expression as they allow for simple and efficient gene expression of T7-based expression vectors (Francis and Page, 2010). Origami and Rosetta-Gami strains can maintain the reducing environments of the bacterial cytosol. Consequently, they aid in the formation of disulfide bonds, thereby greatly enhancing the solubility of folded, active disulfide bond-containing proteins produced (Francis and Page, 2010). SHuffle T7 is an enhanced BL21 derivative designed for improved expression of proteins that require proper disulfide bond formation (Lobstein et al., 2012). We compared these strains for their potential to produce our fusion proteins at various temperatures using SDS-PAGE and Western blot. Interestingly, we found that, for Loxin, all positively transformed strains, except Origami 2, were able to express the fusion protein. In contrast, we received less promiscuous results for the Phoni construct. Only half of the available strains could initially be transformed. This may be reasoned by possible toxic effects resulting in cell death or arrested growth due to protein expression prior to inductions (Francis and Page, 2010). Nevertheless, the two strains, BL21 and SHuffle T7, were found to express the fusion protein at all three temperatures.

Based on our screening, we conclude that several strains are generally suitable for the expression of spider venom enzymes, while Origami 2 and Rosetta-Gami B should be excluded from future studies as they either failed to be transformed or did not produce detectable target protein. The absence of target proteins using Origami 2 may be due to an elevated degradation of the expressed product with bacterial proteases (Francis and Page, 2010). Therefore, we evaluated BL21 and its derivative SHuffle T7 as the most promising strains based on our data and due to their previous successful implementation for the expression of other spider venom components (Klint et al., 2013; Bertelsen et al., 2021; Lüddecke et al., 2023). BL21 has also been used for the expression of a variety of venom enzymes and peptides (Yuan et al., 2004; Turchetto et al., 2017; Najafi et al., 2025). However, it will be a fruitful future development to explore a larger array of readily available specialized expression hosts, such as the CyDisCo system, or even including fungal systems such as *Pichia pastoris* strains (Matos et al., 2014). Although no spider venom enzyme has yet been produced using a fungal system, recombinant snake venom and scorpion venom enzymes have been successfully expressed in *P. pastoris* strains (Zhu et al., 2010; Amorim et al., 2018; Krayem et al., 2022). These systems are characterized by a short doubling time, the ability to introduce post-translational modifications (e.g., glycosylation and disulfide bond formation), and the possibility of secretory expression into the medium. However, the required plasmid amounts in the transformation stage are vastly higher and lead to lower transformants per µg of DNA than those in prokaryotic systems. Furthermore, protein production in yeast systems is more time-consuming (approximately 100 h) and depends on the consumption of methanol during the expression phase, which requires a strong cooling system due to high heat production and precise concentration monitoring due to toxic effects on cell viability. Selection markers, such as antibiotic resistance genes, are limited in yeast systems, which often lead to contamination, and secreted proteases from *P. pastoris* may degrade the target proteins (Karbalaei et al., 2020). Even though fungal systems feature promising advantages for toxin production, their application in venom biotechnology has been scarce to date (Rivera-de-Torre et al., 2022; Lüddecke et al., 2023).

In addition to the host system, expression temperature is an important component of protein expression. The reaction rate–temperature rule predicts that the reaction speed of biochemical processes doubles per 10 °C increase in temperature; hence, higher expression temperatures are correlated with increased protein production. Yet, at the same time, the likelihood for misfolding and the activity of most proteases are elevated, thus increasing the likelihood of autoproteolysis of expression proteins (Francis and Page, 2010). Hence, it is important to unveil the optimal expression conditions for each protein of interest, and accordingly, we devoted part of our study to investigating the optimal temperature range for our proteins. As stated previously, in many instances, both proteins were expressed at multiple temperatures, all of which resulted in insoluble production as inclusion bodies. However, the results obtained demonstrated variations in yield, contingent on incubation time and temperature. Consequently, we propose that the exploration of different expression temperatures is beneficial even in instances where the target protein becomes insoluble.

In light of the above insights on selecting host systems and expression temperatures, our study underpins the importance of probing a diverse set of expression strains and expression conditions, instead of following a one-dimensional default protocol. Had we opted to explore only a single strain (e.g., Origami 2) and a single temperature (e.g., 37 °C, chosen for the highest anticipated yield) instead of our multi-pronged assessment, our expression attempt would have been entirely unsuccessful. To summarize the proven functional systems for spider venom enzyme expression, we present the systems used to date in Table 4.

**TABLE 4:** Summary of spider venom enzyme expression systems. The table provides an overview of previously expressed spider venom enzymes, their species of origin, used expression systems, and fusion partners.

Name	Class	Species of origin	Expression system	Fusion partner	Reference
Phoni	Serine protease	*Phoneutria nigriventer*	BL21 and ST7	His-Tag, Thioredoxin A, and FXa site	This study
Loxin	Astacin	*Loxosceles intermedia*	Ori B, BL21, RG 2, and ST7	His-Tag, Thioredoxin A, and FXa-site	This study
LALP1	Astacin	*Loxosceles intermedia*	BL21	His-Tag and Thrombin-site	Da Silveira et al. (2007b)
LALP3	Astacin	*Loxosceles intermedia*	BL21	His-Tag and SUMO	Morgon et al. (2016)
r*Bv*Hyal	Hyaluronidase	*Brachypelma vagans*	Baculovirus-infected Sf9 cells	-	Clement et al. (2012)
Dietrich’s hyaluronidase	Hyaluronidase	*Loxosceles intermedia*	BL21	His-Tag and Thrombin-site	Ferrer et al. (2013)
LiHyal2	Hyaluronidase	*Loxosceles intermedia*	Baculovirus-infected Sf9 cells	V5-His-Tag	De-Bona et al. (2021)
LiD1	Phospholipase D	*Loxosceles intermedia*	*E. coli* XLOR*L*	β-Galactosidase protein	Kalapothakis et al. (2002)
LiRecDT2 and LiRecDT3	Phospholipase D	*Loxosceles intermedia*	BL21	His-Tag and Thrombin-site	Da Silveira et al. (2006)
LiRecDT4 and LiRecDT5	Phospholipase D	*Loxosceles intermedia*	BL21	His-Tag and Thrombin-site	Da Silveira et al. (2007a)
LiRecDT6	Phospholipase D	*Loxosceles intermedia*	BL21	His-Tag and Thrombin-site	Appel et al. (2008)
LIPLD1 and LIPLD2	Phospholipase D	*Loxosceles laeta*	BL21	His-Tag and SUMO	Catalán et al. (2011)
aIB2bi	Phospholipase D	*Loxosceles arizonica*	BL21	His-Tag	Lajoie et al. (2013)
LgRec1	Phospholipase D	*Loxosceles gaucho*	BL21	His-Tag	Magalhães et al. (2013)
Q1KY80	Phospholipase D	*Loxosceles laeta*	Cell-free (*E. coli*)	-	Paas et al. (2024)

#### 3.8.3 Lessons on refolding and purification

Once the desired protein is successfully expressed as a fusion protein, the final challenge of the expression workflow is its subsequent cleavage and purification to allow subsequent studies on the proteins’ activity and biological role. Although this appears as generally a straightforward process, numerous hurdles can emerge at this stage, and it is important to explore solutions to those.

In our case, the selected proteins were expressed as inclusion bodies, and we had to facilitate their refolding prior to fusion protein cleavage. A range of refolding approaches is available for various protein groups, but at the time of writing, virtually none have been established for spider venom enzymes. Hence, we compared different approaches for our Loxin protein. Our analysis revealed a high influence of pH, buffer chemistry, and protein concentration on the solubilization and activity of the refolded protein. The highest precipitations were observed using pH levels below or near the Loxin pI of 7.7, independent of the utilized initial fusion protein concentration. As proteins possess a net charge of zero at their pI, they are capable of approaching each other with minimal charge repulsion, thereby resulting in aggregation (Burgess, 2009). Compared to this, pH levels above the pI led to lower precipitations even with the highest initial fusion protein concentration of 0.3 g/L. For the second and third dialysis approaches, an initial concentration of 0.3 g/L in combination with a series of varying dialysis buffers was explored. The first dialysis for both approaches was supplemented with high concentrations of L-arginine, leading to no observed precipitations. Further exchange to a zinc-containing dialysis buffer and a buffer containing only Tris resulted in the highest concentration of refolded fusion protein. The mechanism of action of L-arginine remains to be understood in detail, but it is known for its ability to suppress protein aggregation (Tsumoto et al., 2004), while zinc is a necessary cofactor for the activity of astacin-like metalloproteases (Trevisan-Silva et al., 2010).

In theory, we have carefully designed our fusion protein to avoid expression as inclusion bodies and, for this purpose, relied on the chaperone-like activity of thioredoxin A. However, based on our experiments, the fusion protein was absent from the soluble part of lysed cells but present in the insoluble debris (Supplementary Figure S3); therefore, thioredoxin A may be of limited capability to aid in the folding and solvation of the expressed fusion protein. It may, therefore, be an important future consideration to construct fusion proteins using other partners to the selected spider venom enzyme; in particular, the pET-SUMO system may be promising. These vectors allow the addition of a 6x-His-Tag, including a thrombin linker or a SUMO linker (Ferrer et al., 2013; Morgon et al., 2016). The SUMO fusion partner is known for its ability to enhance protein solubility, support the correct folding, and protect against proteolytic degradation of the recombinant protein (Butt et al., 2005; Zuo et al., 2005). Therefore, the utilization of SUMO as a fusion partner should be contemplated for future studies to explore its ability in soluble protein production.

Further experiments discovered the attachment of the Loxin fusion protein on surfaces, leading to protein losses and low FXa cleavage efficacy, resulting in low enzyme yields. The attachment of proteins on solid surfaces is influenced by various properties, such as the hydrophobicity, hydrophilicity, and charge of both the surface and the protein (Tengvall, 2011; Ouberai et al., 2014; Hada et al., 2019). Additionally, the contact time, protein concentration, pH of the solution, and temperature influence the interaction between surfaces and proteins (Ouberai et al., 2014). Although the protein–surface interaction is crucial for processes such as immobilization, it may also hinder further processing. Thus, different storage materials were explored for their ability to prevent surface adsorption of the Loxin fusion protein. Surprisingly, the tested low-binding tubes were found to exert protein–surface interactions, and further treatment with detergents was required to prevent significant losses. The addition of Tween 20 and Triton X-100 in low concentrations yielded the complete solubilization of the Loxin fusion protein. Due to its well-known ability to increase and stabilize enzyme activity at low concentrations, we opted to continue our work with Tween 20-treated material (Duskey et al., 2021). Preventing surface adsorption had a significant impact on FXa cleavage, as indicated by complete cleavage of the Loxin fusion protein after 2–3 days, whereas under previous conditions, cleavage remained incomplete even after 14 days. This lesson underpins the importance of careful consideration of the materials and treatments utilized prior to subjecting fusion proteins to cleavage as it can have dramatic effects on the drawn conclusions.

Finally, for subsequent purification of the target enzyme, we compared affinity and ion exchange chromatography. Chromatographic purification represents a well-established methodological approach that has been applied to a range of venom enzymes (Zhu et al., 2010; Sutti et al., 2014; Amorim et al., 2018; Rivera-de-Torre et al., 2022). First, a non-absorptive IMAC was carried out, revealing an affinity of Loxin toward the IMAC resin. This affinity may result from the positive charge of the target enzyme as the applied pH of 8.0 is below the enzyme’s isoelectric point (9.0) (Belew et al., 1987). Interestingly, cation exchange chromatography at the same pH level revealed no protein binding, while adjustment to a pH of 6.0 yielded high amounts of pure Loxin. Consequently, the adjustment of pH levels in higher magnitudes may help the achievement of an adequate purification performance (Kopaciewicz and Regnier, 1983). Given that ion exchange chromatography is also a relatively mild approach to chromatographic separation, which is an important consideration for large and sensitive proteins such as enzymes, we recommend this technology for the efficient purification in future work.

## 4 Conclusion

In this study, we explored the recombinant expression of spider venom enzymes, focusing on the technical details and potential pitfalls encountered during the expression and purification process development. Previous findings have highlighted the substantial potential of spider venom enzymes as a resource for biotechnological applications. In this study, we identified key challenges associated with the expression of these enzymes in *Escherichia coli*, particularly regarding the formation of inclusion bodies and the subsequent need for effective refolding strategies. Through systematic evaluation of various expression strains and conditions, we determined favorable conditions for the production of the target protein. We also demonstrated the importance of optimizing purification protocols, including immobilized metal affinity chromatography and cation exchange chromatography, to achieve a successful isolation of the target enzyme. Furthermore, the prevention of surface absorbance and the influence of pH levels on the solubility of proteins were examined. The results of this study not only provide a methodological framework for future research in the field of arachnid toxinology but also emphasize the need for further exploration of the diverse expression conditions and systems for heterologous spider venom enzyme biosynthesis. By continuing to refine recombinant expression techniques and purification methods, we can unlock the potential of these enzymes for various applications in medicine, biotechnology, and beyond. Overall, our research contributes to a deeper understanding of spider venom enzymes and sets the stage for their future characterization and utilization in diverse biotechnological settings.

## Data Availability

The original contributions presented in the study are included in the article/Supplementary Material; further inquiries can be directed to the corresponding authors.
