# Supplementary material for: Prospects and challenges of recombinant spider venom enzymes: insights from Loxosceles and Phoneutria venom protease expressions
Source: Front Bioeng Biotechnol. 2025 Sep 19;13:1668774. doi: 10.3389/fbioe.2025.1668774 (PMC12492492; doi:10.3389/fbioe.2025.1668774)
Supplement: Supplementary file 1 [file DataSheet1.zip › Supplements/Supplementary_Table_S1.docx]

**Table S1:** Dialysis approaches tested for refolding of Loxin fusion protein, using different buffers, pH and initial fusion protein concentrations

| **Dialysis approach** | **Buffer** | **pH** | **Initial fusion protein [g/L]** |
| --- | --- | --- | --- |
| First dialysis approach | - TRIS NaCl (20 mM TRIS, 150 mM NaCl) | 7.0, 7.5, 8.5, 9.0 | 0.1, 0.2, 0.3 |
|  | - HEPES (20 mM) | 6.8 | 0.1 |
|  | - MES (20 mM) | 6.5 | 0.1 |
| Second dialysis approach | - TRIS NaCl (20 mM TRIS, 150 mM NaCl)   supplemented with  1.round: L-arginine  2.round: zinc sulfate  3.round: none | 8.5 | 0.3 |
| Third dialysis approach | - TRIS (50 mM)   supplemented with  1.round: L-arginine  2.round: zinc sulfate  3.round: none | 8.5 | 0.3 |
