# Supplementary figures and images for: Prospects and challenges of recombinant spider venom enzymes: insights from Loxosceles and Phoneutria venom protease expressions
Source: Front Bioeng Biotechnol. 2025 Sep 19;13:1668774. doi: 10.3389/fbioe.2025.1668774 (PMC12492492; doi:10.3389/fbioe.2025.1668774)

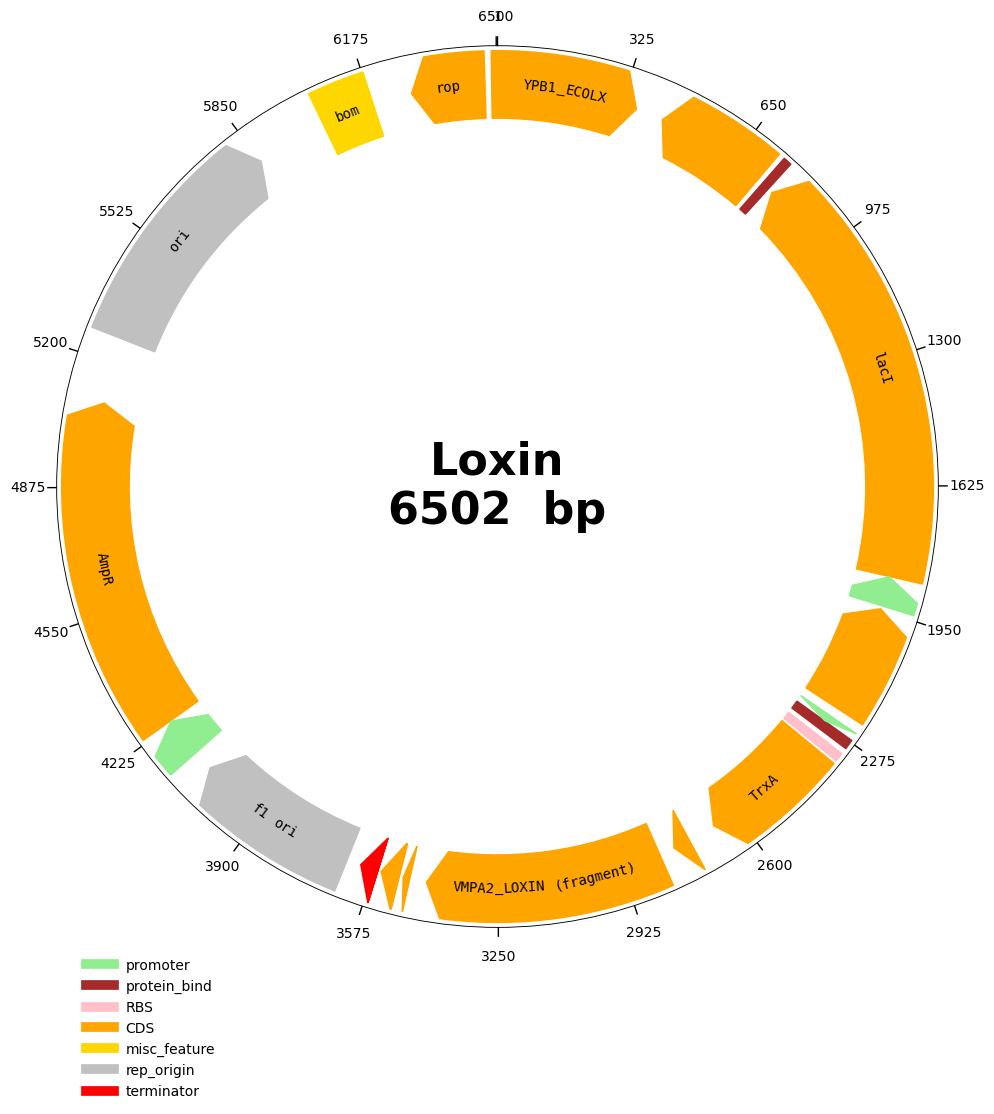

Supplement: Supplementary file 1 [file DataSheet1.zip › Supplements/S1_NanoporeLoxin.png]

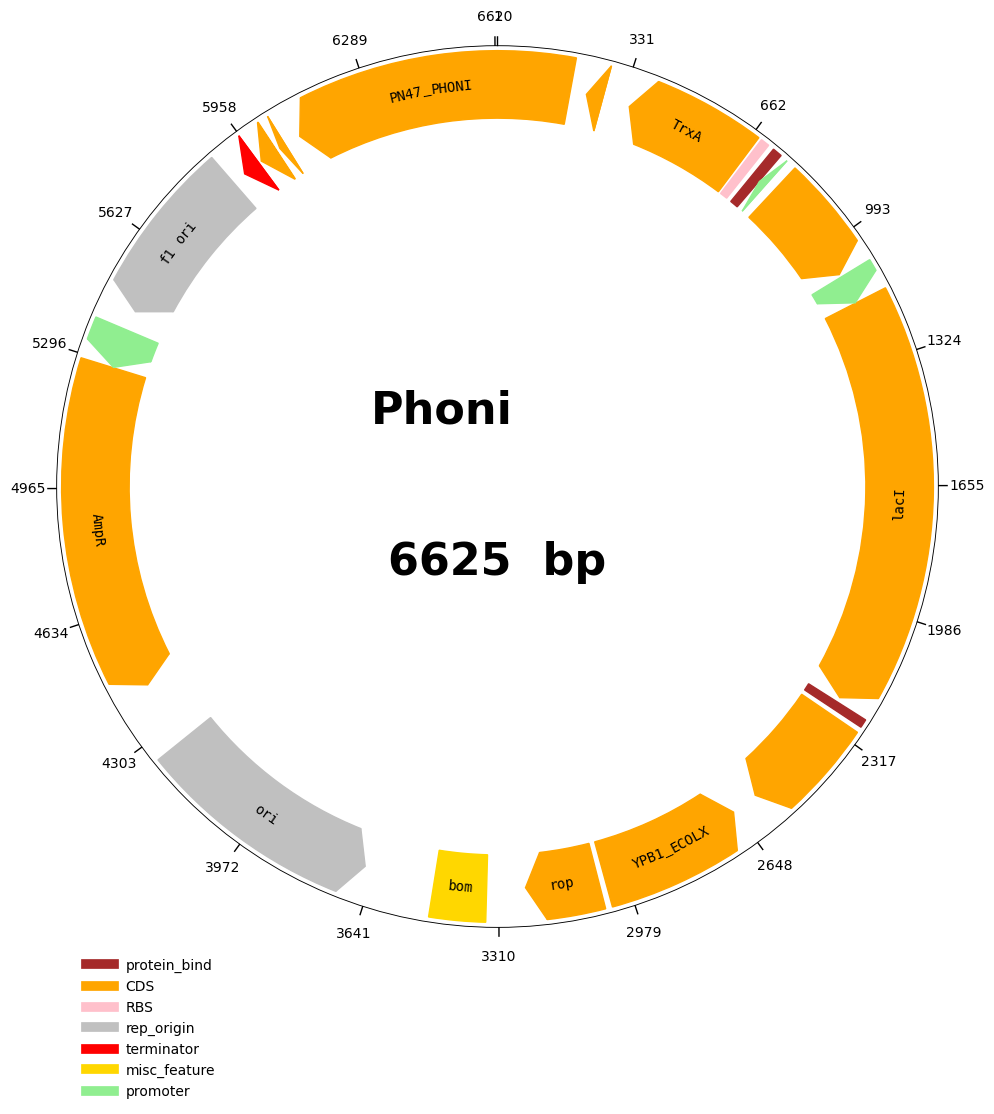

Supplement: Supplementary file 1 [file DataSheet1.zip › Supplements/S2_NanoporePhoni.png]

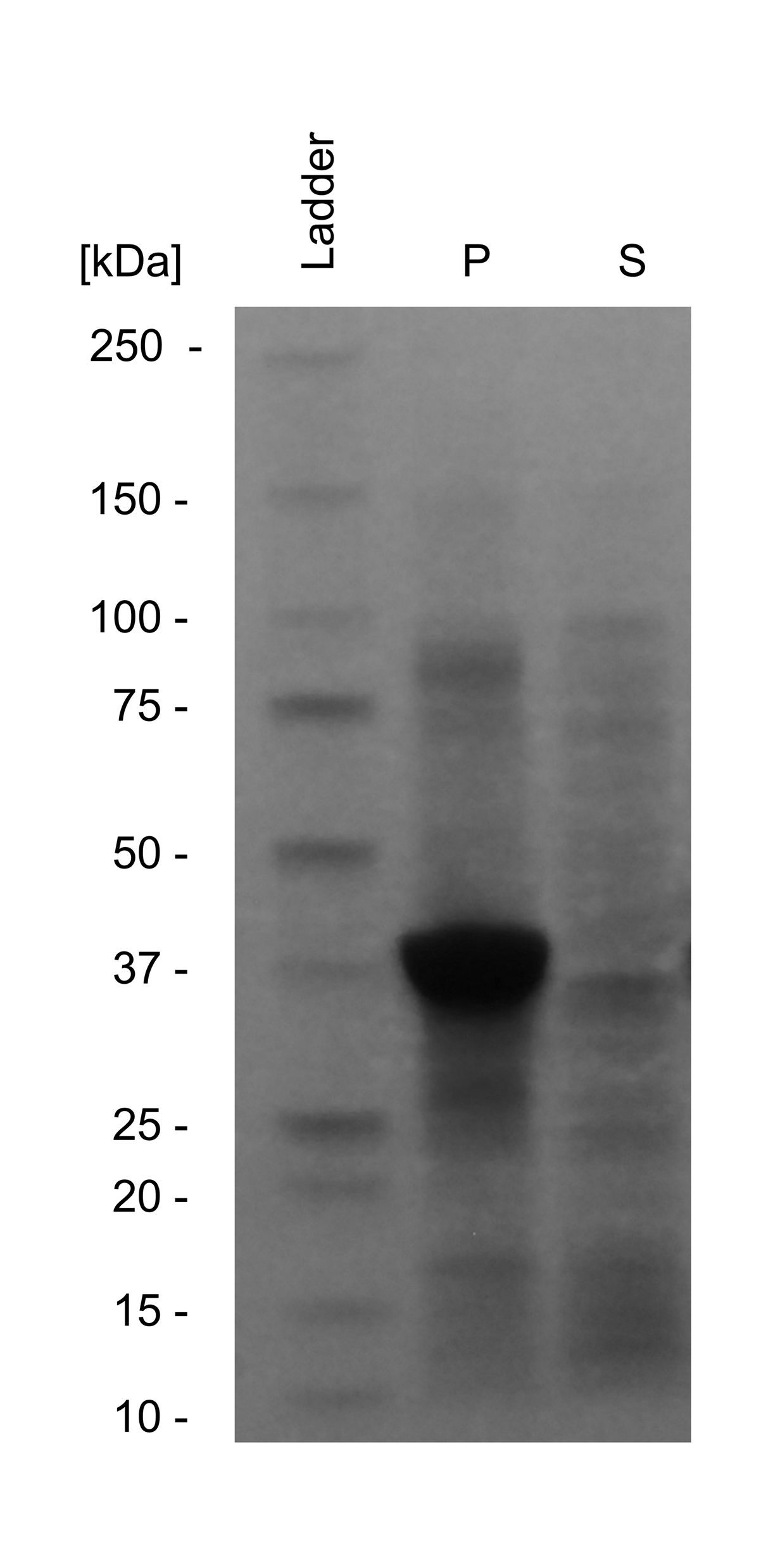

Supplement: Supplementary file 1 [file DataSheet1.zip › Supplements/S3_InclusionBodies.png]

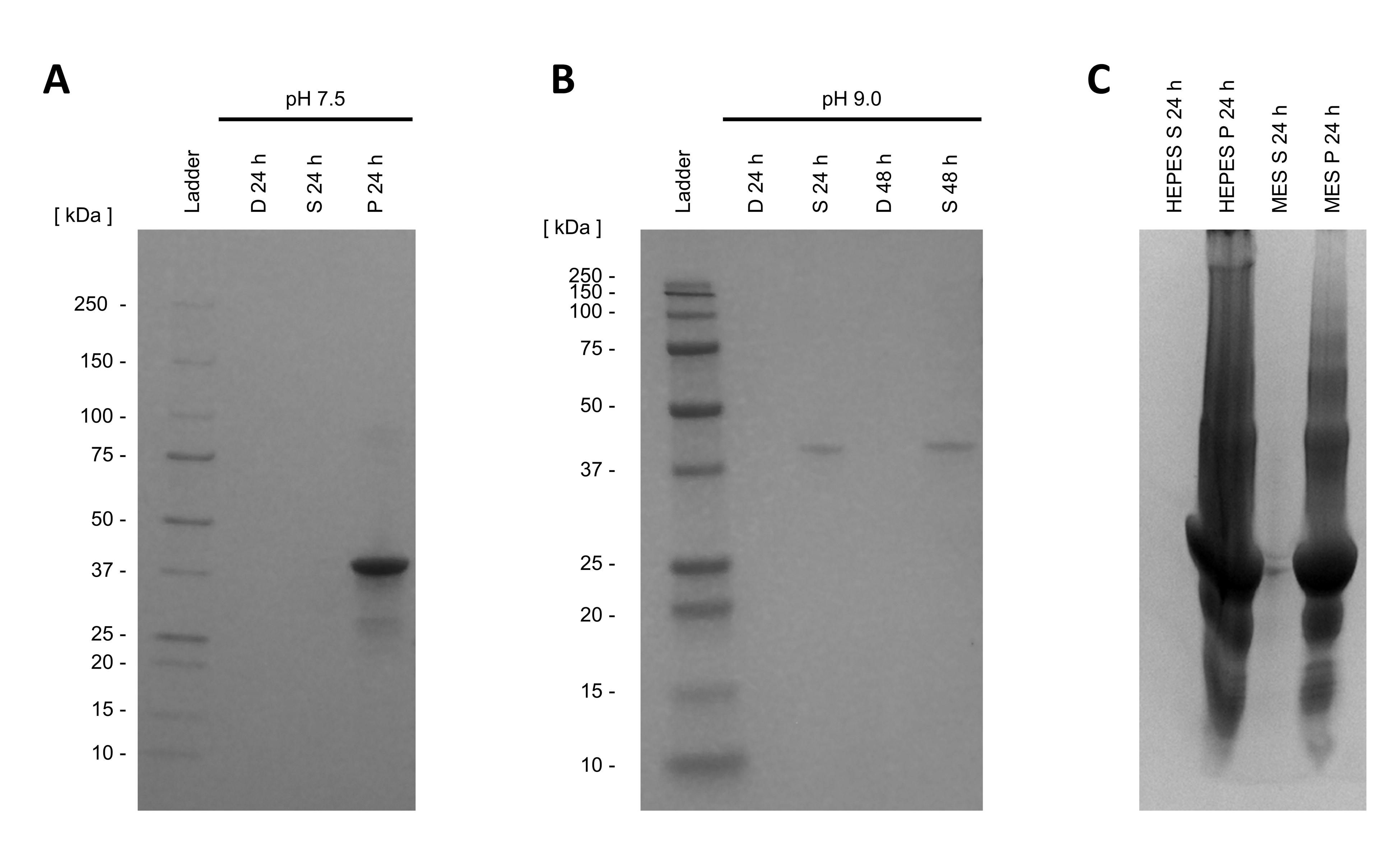

Supplement: Supplementary file 1 [file DataSheet1.zip › Supplements/S5_refolding.png]

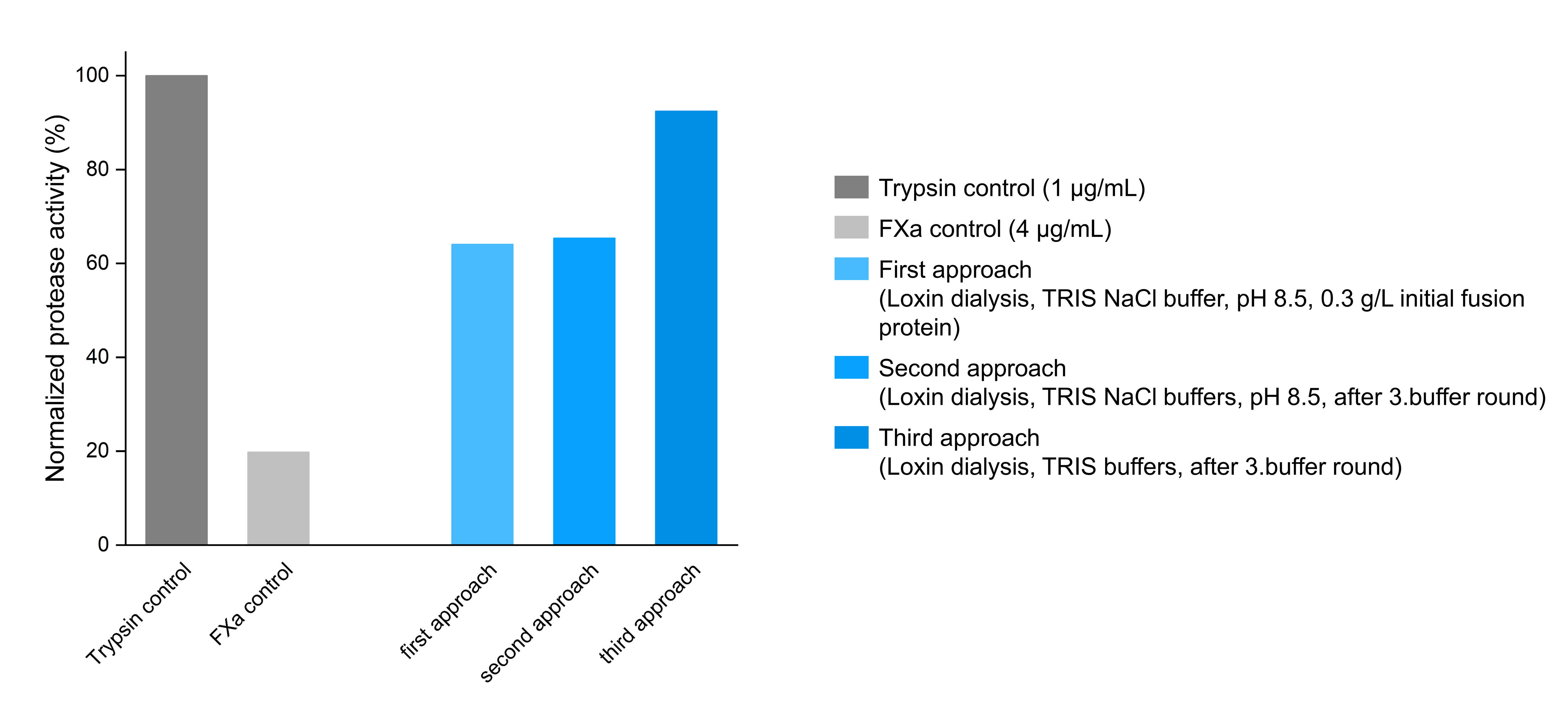

Supplement: Supplementary file 1 [file DataSheet1.zip › Supplements/S6_protease_activity.png]
